# Methicillin resistant *Staphylococcus aureus* in the United Arab Emirates: a 12-year retrospective analysis of evolving trends

**DOI:** 10.3389/fpubh.2023.1244351

**Published:** 2023-12-07

**Authors:** Jens Thomsen, Najiba M. Abdulrazzaq, The UAE AMR Surveillance Consortium, Godfred Antony Menezes, Carole Ayoub Moubareck, Dean B. Everett, Abiola Senok

**Affiliations:** ^1^Department of Environmental and Occupational Health and Safety, Abu Dhabi Public Health Center, Abu Dhabi, United Arab Emirates; ^2^Department of Pathology and Infectious Diseases, College of Medicine and Health Sciences, Khalifa University, Abu Dhabi, United Arab Emirates; ^3^Al Kuwait Hospital Dubai, Emirates Health Establishment, Dubai, United Arab Emirates; ^4^Public Health Sector, Ministry of Health and Prevention, Dubai, United Arab Emirates; ^5^Department of Medical Microbiology and Immunology, RAK Medical and Health Sciences University, Ras Al Khaimah, United Arab Emirates; ^6^College of Natural and Health Sciences, Zayed University, Dubai, United Arab Emirates; ^7^Biotechnology Center, Khalifa University, Abu Dhabi, United Arab Emirates; ^8^Infection Research Unit, Khalifa University, Abu Dhabi, United Arab Emirates; ^9^College of Medicine, Mohammed Bin Rashid University of Medicine and Health Sciences, Dubai, United Arab Emirates; ^10^School of Dentistry, Cardiff University, Cardiff, United Kingdom

**Keywords:** methicillin resistant *Staphylococcus aureus*, antimicrobial resistance, United Arab Emirates, national surveillance, Arabian Gulf region, MRSA

## Abstract

**Introduction:**

Methicillin resistant *Staphylococcus aureus* (MRSA) is a major contributor to the global burden of antimicrobial resistance (AMR). As MRSA continues to evolve, the need for continued surveillance to evaluate trends remains crucial. This study was carried out to assess MRSA trends in the United Arab Emirates (UAE) based on analysis of data from the national AMR surveillance program.

**Methods:**

We carried out a 12-year (2010–2021) retrospective analysis of MRSA demographic and microbiological data collected as part of the UAE national AMR surveillance program. Participating centers from across the country routinely submit AMR surveillance data collected by trained personnel to the National AMR Surveillance Committee, where data is analyzed using a unified WHONET platform. Data on non-duplicate isolates associated with clinical infections were obtained and included in the analysis.

**Results:**

A total of 29,414 non-duplicate MRSA isolates associated with clinical infections were reported between 2010 and 2021 (2010: *n* = 259; 2021: *n* = 4,996). MRSA represented 26.4% of all *S. aureus* (*n* = 111,623) isolates identified during the study period. In 2010, among the *S. aureus* isolates with reported oxacillin testing, 21.9% (*n*/*N* = 259/1,181) were identified as MRSA and this showed an increase to 33.5% (*n*/*N* = 4,996/14,925) in 2021. Although there was variation in the distribution of MRSA across the seven emirates of the country, most had an upward trend. Patient demographics reflected a male preponderance, with most being adults and from the outpatient setting. Isolates were mostly from skin and soft tissue infection specimens (72.5%; *n*/*N* = 21,335/29,414). Among the inpatients (*N* = 8,282), a total of 3,313 MRSA isolates were from specimens obtained ≤ 48 h after admission indicative of community acquired infection. Increasing resistance trends were observed for most antibiotics including ciprofloxacin, levofloxacin, moxifloxacin, erythromycin, gentamicin, trimethoprim-sulfamethoxazole, and quinupristin/dalfopristin. Low levels of resistance (0.0–0.8%) were sustained for linezolid except for 2015, 2016, and 2017 with 2.5, 2.6, and 2.9%, respectively. No confirmed vancomycin resistance was reported.

**Conclusion:**

The increasing trend of MRSA isolates associated with clinical infections in the hospital and community settings is a concern. Continued monitoring including incorporation of genomic surveillance and infection control measures are recommended to stem the dissemination.

## 1 Introduction

Antimicrobial resistance (AMR) is of significant concern globally as infections caused by resistant pathogens are associated with significant patient morbidity and mortality as well as increased healthcare costs (1). There is also a concern that the increased utilization of antibiotics in COVID-19 patients due to co-infections and the widespread use of azithromycin in the early days of the pandemic may result in a worsening of the global AMR crisis (2, 3) with a call for close monitoring of national and global AMR trends. Since the first identification of methicillin resistant *Staphylococcus aureus* (MRSA) in the United Kingdom in the early 1960s, MRSA has disseminated globally and is an important cause of nosocomial infections contributing to the burden of AMR in many countries (4, 5). The molecular epidemiology of MRSA has remained dynamic with an evolution toward increasing predominance of community associated MRSA lineages (CA-MRSA) in nosocomial infections (4, 6).

The United Arab Emirates (UAE) is located in the Arabian Peninsula and is a federation of seven emirates namely Abu Dhabi, Dubai, Sharjah, Ras Al Khaimah, Umm Al-Quwain, Fujairah, and Ajman. The country has a highly developed economy, is known for its modern infrastructure and is a cosmopolitan setting being home to expatriates from over 200 nations. The UAE is a global hub for commerce, trade, and tourism. This dynamic population movement might facilitate the introduction of drug-resistant pathogens into the country's community and hospital settings thus contributing to the burden of infections and AMR trends.

Findings from a single center study in the UAE, reported *S. aureus* in the majority of patients with skin and soft tissue infections with MRSA detection in 23% of culture-positive patients (7). In addition, *S. aureus* has been shown to contribute to the burden of co-infections among hospitalized COVID-19 patients in the UAE (8). Recently, molecular characterization of MRSA isolates associated with clinical infections in the UAE revealed the presence of wide clonal diversity as well as identification of rare and novel variant strains (9, 10). In addition, available data also indicates that CA-MRSA lineages have overtaken hospital acquired MRSA (HA-MRSA) lineages as aetiological agents of nosocomial infections in the UAE (9, 11). Therefore, with the indication that MRSA contributes to the burden of AMR in the UAE and the reported shifts in the molecular epidemiology of MRSA there is a need for an understanding of MRSA trends in the UAE. Therefore, this study was carried out to assess MRSA trends including prevalence and antibiogram patterns in the UAE based on retrospective analysis of data from the national AMR surveillance program.

## 2 Methods

This study is a retrospective data analysis of MRSA data from the UAE for the 12-year period 2010–2021. MRSA trends were assessed by analysis of routinely collected national level AMR surveillance data. This includes data on overall burden of *S. aureus* infections and including those caused by isolates identified as MRSA.

### 2.1 Data collection

The national AMR data is collected from a network of participating healthcare facilities (hospitals, centers, and clinics) and diagnostic laboratories across the country. These include primary, secondary and tertiary care facilities across governmental and private healthcare sectors. Participation of sites in the national AMR Surveillance program is voluntary and no financial incentives are offered. All data are collected from routine patient care, cleaned, and analyzed using a unified platform (WHONET)^1^ as described by Thomsen et al. (12). Training on data collection is provided to ensure quality assurance, standardization, and accuracy. The fully anonymized data includes demographic data (age, gender, nationality, hospital site/location etc.), clinical and microbiological data such as specimen source, specimen date, and antibiogram.

### 2.2 Bacterial identification and antimicrobial susceptibility testing

The participating centers used at least one commercial, automated system for bacterial identification and antimicrobial susceptibility testing. These automated systems include VITEK^®^ (BioMérieux SA, Craponne, France), BD Phoenix™ (Becton Dickinson, New Jersey, USA) and MicroScan WalkAway (Beckman Coulter, Brea, CA, USA) and were used in conformity with manufacturer guidelines. Only one laboratory relied solely on a manual system for bacterial identification using API^®^ (Analytical Profile Index. BioMérieux SA, Craponne, France). Two laboratories used manual antimicrobial susceptibility testing methods (disc diffusion/Kirby Bauer). For the reporting of antimicrobial resistance, CLSI breakpoints were routinely applied by reporting sites and at the central level to determine susceptibility profiles of isolates (13).

### 2.3 Statistical analysis

Data analysis was routinely carried out using the WHONET 2023 software. For additional statistical analysis other software packages used were IBM SPSS Statistics, version 29.0 (IBM SPSS Software), and Epi Info^TM^ for Windows v7.2.4.2022, Centers for Disease Control and Prevention. Statistical significance of temporal trends for antimicrobial resistance percentages was calculated if data from at least 5 years was available. If fewer than 30 isolates per year were reported, or data was not available for all years within the considered period, trend analysis was not conducted. Statistical significance of trends is expressed as a *p*-value, calculated by a Chi-square for trend test (extended Mantel-Haenszel), using SPSS or Epi Info™. For testing the statistical significance of the difference for mortality and ICU admission a Chi^2^-test was used, for testing the statistical significance of the difference for length of stay the non-parametric weighted Log-rank test was used. A *p*-value of < 0.05 was considered statistically significant.

## 3 Results

### 3.1 Distribution of reporting sites for national AMR surveillance

**Figure 1:**
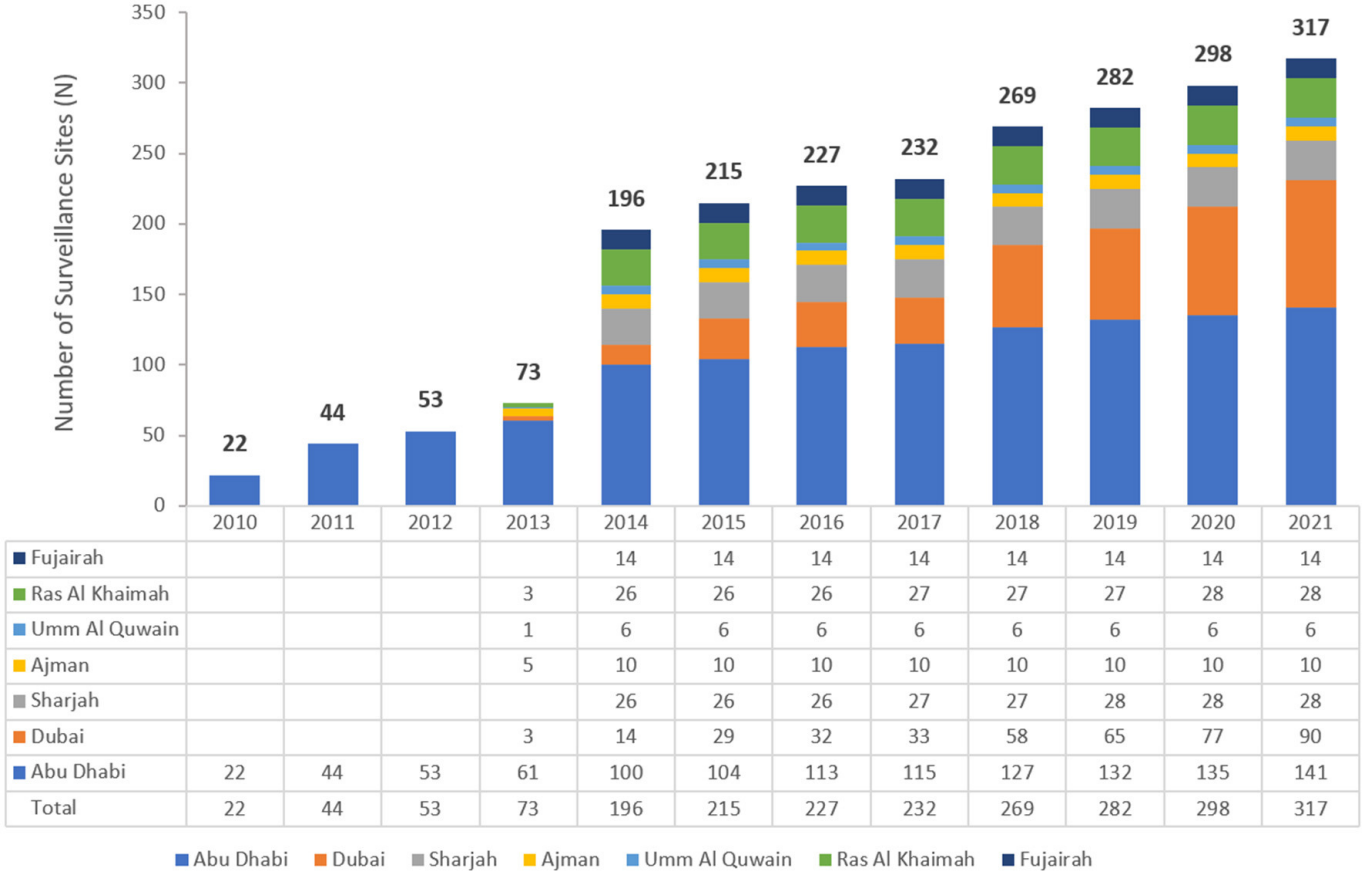
Number of surveillance sites per year and Emirate (2010–2021).

The number of reporting sites increased during the early implementation phase of the national AMR surveillance program, from 22 in 2010 to 317 in 2021 (Figure 1). These comprised of primary, secondary, and tertiary care facilities (87 hospitals, 230 centers/clinics) as well as 45 diagnostic laboratories across both the public and private health sectors. The national AMR system is considered largely representative of the whole healthcare system in the UAE, representing approximately 57.6% of all 156 hospitals, and 8.5% of all 2,730 ambulatory healthcare centers/clinics in the UAE. From 2014 to 2021, participating centers were from all seven emirates in the country, in contrast to 2010–2012 where the centers were all from Abu Dhabi emirate and 2013 when they were from only five emirates.

### 3.2 Bacterial population and demographic distribution

**Figure 2:**
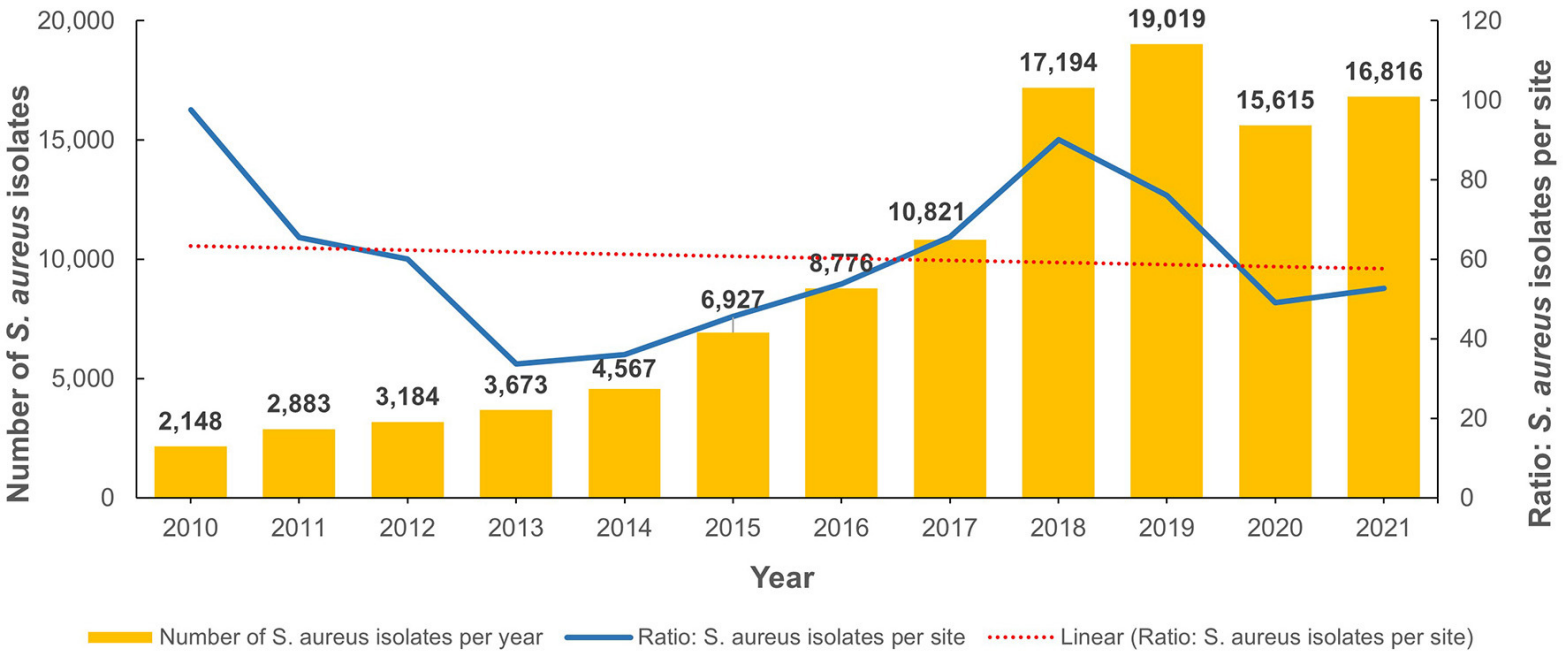
Distribution of *Staphylococcus aureus* isolates (2010–2021).

From 2010 to 2022, the total number of reported non-duplicate *S. aureus* isolates was 111,623. Figure 2 shows the annual distribution trends as well as the number of isolates per site when normalized for the increased number of reporting sites per annum. Of these, 29,414 were MRSA isolates and represented 26.4% of all *S. aureus* isolates identified during the study period. The number of *S. aureus* and MRSA isolates for each emirate is shown in Supplementary Table 1A. Table 1 shows the demographic distribution of the patients from whom the MRSA isolates were obtained. Among patients with available data, there was a male preponderance, majority were adults, and they were mainly from the outpatient setting (Table 1). Among the inpatients (*n* = 8,282), a total of 3,313 MRSA isolates were from specimens obtained ≤ 48 h after admission indicative of community acquired infection. The majority of isolates were from specimens from skin and soft tissue infections (72.5%; *n*/*N* = 21,335/29,414; Table 2).

**Table 1:** Demographic distribution of patients with MRSA isolates.

**Number of patients (*****N*** = **29,414)**		**Percentage**
**Gender**		
Male	10,841	36.9
Female	7,347	25.0
Unknown	11,226	38.1
**Age group**		
Pediatric	4,764	16.2
Adult	13,155	44.7
Unknown	11,495	39.1
**Nationality**		
Emirati	5,238	17.8
Non-Emirati	10,796	36.7
Unknown	13,380	45.5
**Hospital location**		
Inpatient	8,282	28.1
Outpatient	11,342	38.6
Unknown	9,790	33.3

**Table 2:** Specimen sources of MRSA isolates.

**Specimen source**	**Number of isolates (*N* = 29,414)**	**Percentage**
Skin and soft tissue	21,335	72.5
Respiratory tract	3,761	12.8
Urine	1,193	4.1
Blood	932	3.2
Genital tract	825	2.8
Others	1,368	4.7

**Figure 3:**
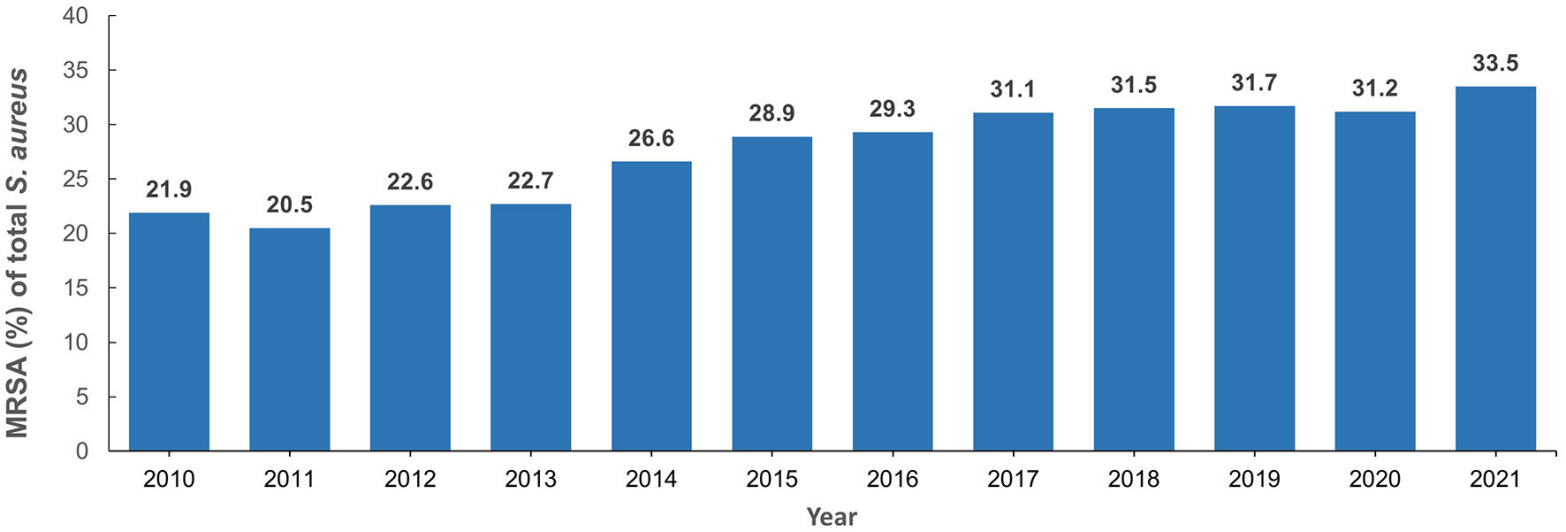
Methicillin resistant *Staphylococcus aureus* prevalence trends (2010–2021).

**Figure 4:**
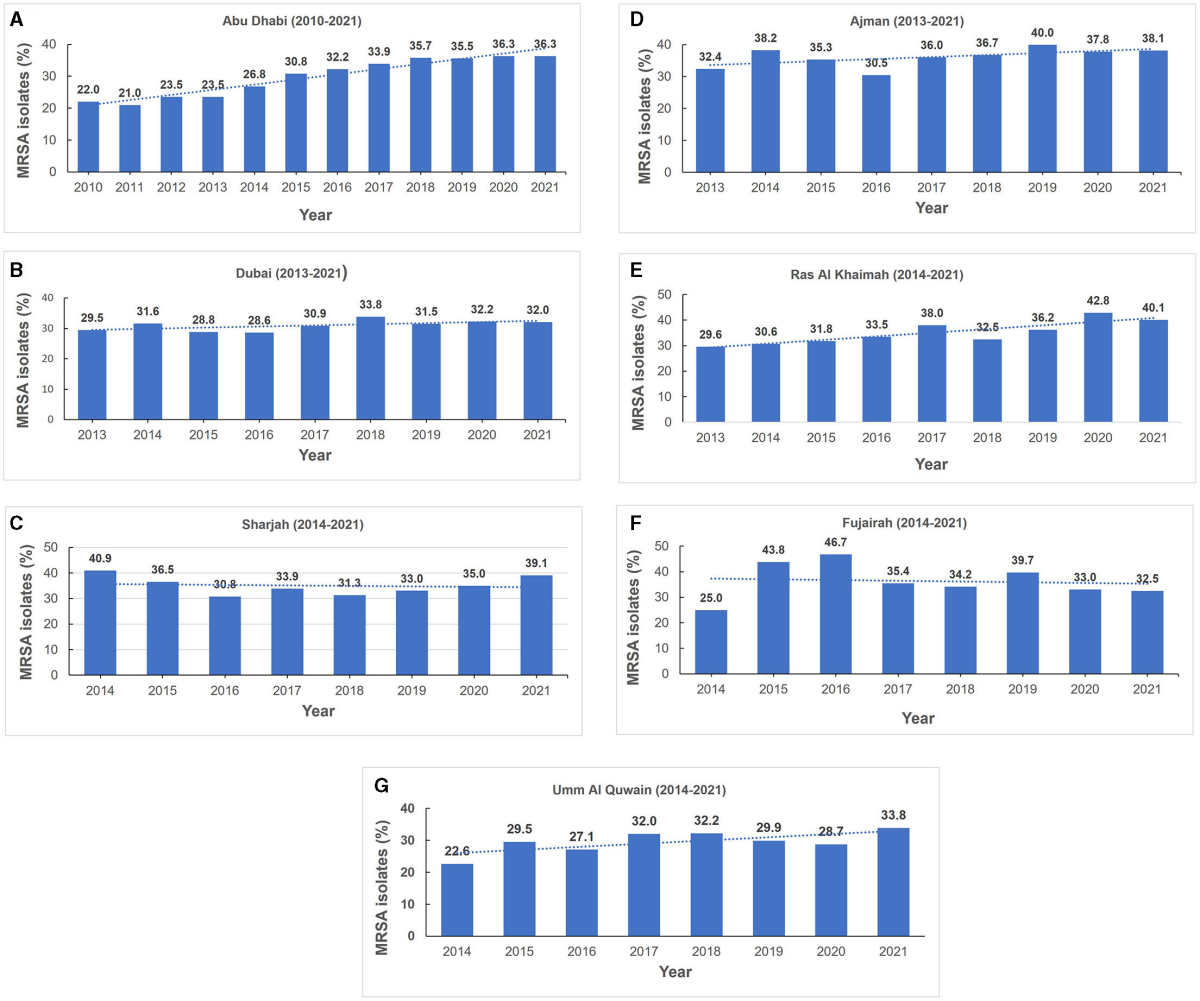
**(A–G)** Annual methicillin resistant *Staphylococcus aureus* prevalence trends (MRSA), by Emirate (2010–2021).

The total number of MRSA isolates reported was 259 in 2010, increasing to 4,996 in 2021 which reflects the increasing number of reporting sites over the surveillance period (Supplementary Figure 1). In 2010, among the *S. aureus* isolates with reported data for oxacillin testing, 21.9% (*n*/*N* = 259/1,181) were identified as MRSA and this showed an increase to 33.5% (*n*/*N* = 4,996/14,925) in 2021 (Figure 3 shows the annual trend). The distribution of MRSA across the seven emirates of the country showed a largely similar upward trend for MRSA prevalence. The highest recorded prevalence of 43.8 and 46.7% in 2015 and 2016, respectively, was from Fujairah, however it should be noted that a downward trend has been observed in this emirate in recent years (Figure 4).

### 3.3 Antimicrobial susceptibility trends

**Figure 5:**
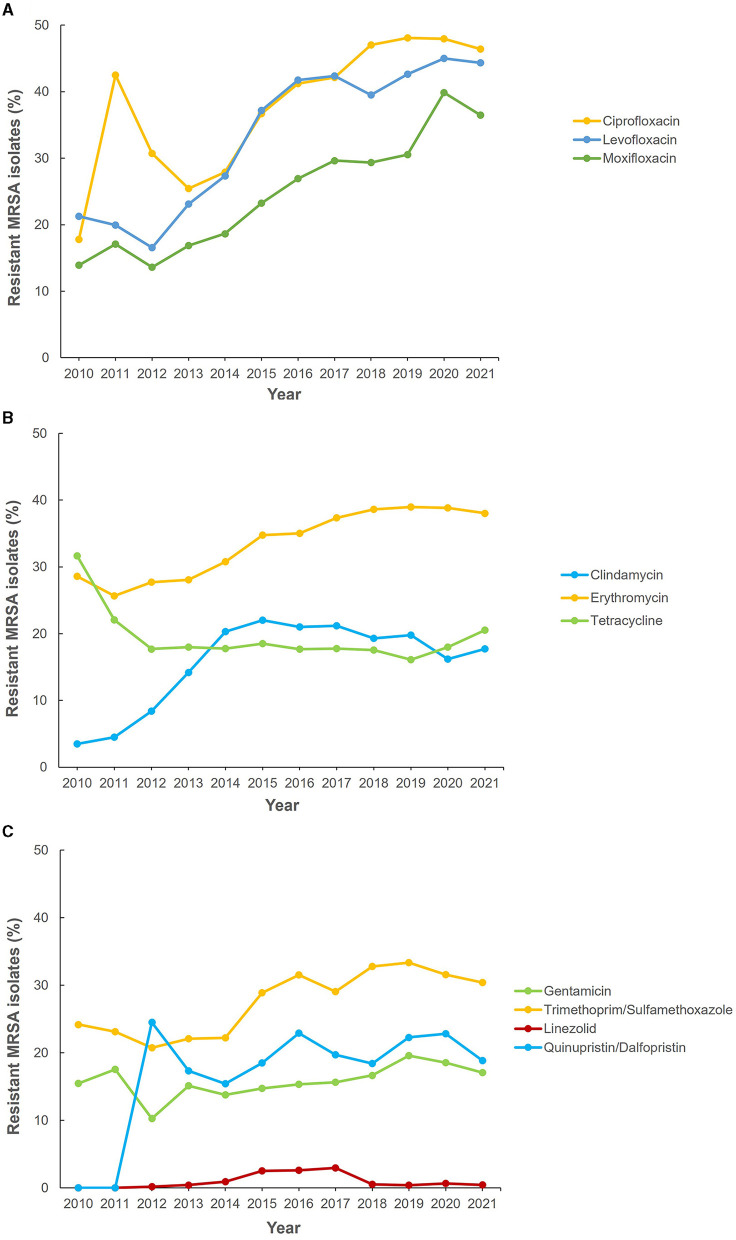
Antibiotic resistance trends for methicillin-resistant Staphylococcus aureus (MRSA) (2010–2021). **(A)** Fluoroquinolones, **(B)** macrolides, and **(C)** other antibiotics.

Figure 5 shows the reported antimicrobial resistance trends of MRSA to various antibiotics during the data collection period. From 2013 to 2020, a significant increment in resistance trends was observed for fluoroquinolones (ciprofloxacin, levofloxacin, and moxifloxacin) (Figure 5A). Among the macrolides, erythromycin showed the highest levels of resistance with an upward trend and over 30% of MRSA isolates being resistant since 2014 (Figure 5B). However, for clindamycin there was a sustained upward resistance trend from 2010 to 2015, followed by a slight decline until 2020 and upward trajectory in 2021 (Figure 5B). Tetracycline resistance decreased between 2010 and 2012 and remained at a low level (< 20%) until 2019 before showing an upward trend from 2019 to 2021 (Figure 5B). An upward pattern of level of resistance was shown for trimethoprim-sulfamethoxazole and gentamicin (2013–2019) with both antibiotics showing a recent downward trend from 2020 to 2021 (Figure 5C). Fluctuation in resistance trend was observed across the years for quinupristin/dalfopristin resistance. For linezolid, there was a sustained low level of resistance during the study period (Figure 5C). Apart from 2015, 2016 and 2017 when the percentage of MRSA isolates resistant to linezolid were 2.5, 2.6, and 2.9%, respectively, for all other years the resistance level was sustained at under 1% of isolates (0.0–0.8%). No confirmed vancomycin resistance was reported.

### 3.4 Outcome analysis

For inpatients, the mean length of stay in hospital for patients with methicillin sensitive *S. aureus* (MSSA) isolates was 10.48 which was significantly lower compared to 12.64 days for those with MRSA isolates (*p* < 0.001). The risk of ICU admission increased by 13.5% (RR: 1.1349, 95% C.I. [1.0664, 12,078]) with MRSA infection which was statistically significant (*p* < 0.001). However, for inpatients where clinical outcome data was available, similar mortality outcome was seen in MSSA (8.2%; *n*/*N* = 808/9,844) and MRSA (8.2%; *n*/*N* = 364/4,434) infections.

## 4 Discussion

The global dissemination and burden of infection associated with MRSA continues to be of concern and understanding the epidemiological trends is crucial for implementing robust infection control strategies. As it has been shown that variations in MRSA epidemiology exist across geographical regions (5, 14), the need for national surveillance data to guide development of appropriate policies is also very important. In this report, we present the findings of the trends in MRSA epidemiology and resistance trends in the UAE based on 12 years of national AMR surveillance data. The findings indicate an upward trend in the burden of MRSA infections as MRSA isolates reported increased from 21.9% in 2010 to 33.5% in 2021. This is in keeping with MRSA prevalence rates in the Arabian Gulf region which range from 15 to 55% as shown by Al-Saleh et al. (15) in a recently published systematic review. It should be highlighted that these prevalence rates were derived from reported data from studies with single or a limited number of participating healthcare facilities with the notable absence of longitudinal national surveillance data (15). Our findings represent the first longitudinal national surveillance MRSA data from the Arabian Gulf region and provides an insight into MRSA trends in the UAE. Such data is pertinent in light of the dynamic population movement and cosmopolitan nature of the country and addresses an important gap in the literature about the burden of MRSA in the Arabian Gulf region. Hence, the increasing trend of MRSA prevalence is of concern particularly in the light of ongoing implementation of infection control strategies. However, our findings are in contrast to lower MRSA detection rates and declining trends which have been reported from other geographical regions (16–19). MRSA infections were historically associated with healthcare settings, primarily affecting patients with co-morbidities and those exposed to invasive medical procedures. However, a significant shift has occurred with the emergence of community-associated MRSA (CA-MRSA) lineages which have becoming increasingly prevalent and are driving nosocomial infections in the hospital setting (20–22). This expansion into the community has raised concerns due to the potential for rapid transmission and limited treatment options. Our findings demonstrate high occurrence of community associated MRSA infections in the UAE which aligns with global trends as well those reported from other countries in the Arabian Gulf region (15, 23, 24). In addition, molecular characterization of MRSA isolates from UAE, Kuwait, Oman and Saudi Arabia have demonstrated a predominance of CA-MRSA lineages harboring SCC-*mecA* types IV, V, VI in both the community and hospital settings (15, 24–26).

The most common source of MRSA isolates were skin and soft tissue infections which is in keeping with reported data from the UAE and across the Arabian Gulf region (7, 9, 15, 25, 27). This finding is particularly pertinent as carriage of the gene encoding for Panton-Valentine Leukocidin (PVL) toxin, a virulence factor associated with *S. aureus* skin and soft tissue infections (SSTI) has been shown to be prevalent among MRSA isolates in our setting (9, 28, 29). A lateral flow test for rapid detection of PVL in *S. aureus* isolates from SSTI was recently reported (30). Based on the current findings of high occurrence of SSTI MRSA in in the UAE, the incorporation of such a test in diagnostic practice is recommended. This will support clinicians in opting for non-pharmacological interventions such as incision and drainage particularly in the management of mild SSTI, thus reducing antibiotic utilization and ultimately the pressure of resistance selection.

MRSA isolates strains frequently exhibit resistance to other classes of antibiotics thus posing further challenges in treatment (5). Sustained high rates of resistance to quinolones and macrolides were demonstrated during our data collection period. These findings align with data from studies in other countries particularly those from the Arabian Gulf region where similar or higher resistance rates have been reported depending on the study setting (15). In contrast, resistance to linezolid was mostly under 1% except for the period from 2015 to 2017. In the recently published systematic review of 39 articles published between 2011 and 2021 from the Arabian Gulf region (15), none of the studies reviewed reported detection of linezolid resistance. Therefore, although our findings indicate very low linezolid resistance rates, it is nevertheless still a call for heightened vigilance and judicious utilization to ensure that we preserve this antibiotic. Vancomycin has been a reliable antibiotic for the treatment of MRSA infections and the potential emergence of vancomycin-intermediate MRSA (VISA) and vancomycin-resistant MRSA (VRSA) strains is a concern. It is therefore noteworthy that neither confirmed VISA nor VRSA isolates were detected in this study. This is also in alignment with data from molecular characterization studies where vancomycin resistance genes were not detected in MRSA isolates from the UAE (9, 10).

The occurrence of missing data observed in the dataset could be related to technical issues arising from differences in electronic health information systems and laboratory platforms across reporting sites. Strategies such as unification of electronic health and laboratory platforms coupled with continued provision of training to personnel could be useful for addressing this. Currently genomic data is not part of the national surveillance dataset, and this is a limitation particularly as there is a paucity of data on the molecular characterization of MRSA strains in the UAE. Currently available literature showed an extensive MRSA repertoire with wide clonal diversity and ongoing emergence of novel variants in the UAE which suggests an evolving MRSA landscape (9, 10). Therefore, to bridge this gap, we advocate for inclusion of genomic data as part of the national MRSA surveillance in the UAE. This will provide much needed insight into the changing molecular landscape of MRSA and support the development of targeted strategies including infection prevention measures. This is crucial in curtailing MRSA trends and alleviating the burden of MRSA infections on the healthcare system.

## 5 Conclusion

In conclusion, the findings from this study show an increasing trend of MRSA isolates associated with clinical infections in the UAE. Continued surveillance with incorporation of genomic data and infection control measures are recommended to stem the continued dissemination.

## Data availability statement

The national AMR Surveillance database managed by the UAE Ministry of Health and Prevention (MOHAP) contains confidential health information, and as such requests to access the datasets should be directed to the UAE Ministry of Health and Prevention (www.mohap.gov.ae).

## Ethics statement

The studies involving humans were approved by Ministry of Health and Prevention Research Ethics Committee (MOHAP/DXB-REC/J.J.J./No. 86/2023), Dubai Scientific Research Ethics Committee (DSREC-GL17-2023). Abu Dhabi Health Research and Technology Ethics Committee (DOH/ZHCD/2023/1316). The studies were conducted in accordance with the local legislation and institutional requirements. Written informed consent for participation was not required from the participants or the participants' legal guardians/next of kin in accordance with the national legislation and institutional requirements.

## Author contributions

AS, JT, NA, GM, CA, and DE: conceptualization and data interpretation. AS, JT, NA, GM, CA, DE, and The UAE AMR Surveillance Consortium: data collection and manuscript review and editing. AS and JT: formal analysis. AS: manuscript preparation. All authors have read and agreed to the published version of the manuscript.

## Figures and Tables

**Table:** 

**Nr**.	**Name**	**Institution**
1	Ahmed Elhag Ahmed	UAE University, College of Medicine and Health Sciences, Al Ain
2	Ahmed F. Yousef	Department of Biology, Center for Membranes and Advanced Water Technology, Khalifa University, Abu Dhabi
3	Amna AlBlooshi	Purelab, Al Ain
4	Dr. Adnan Alatoom	Sheikh Shakhbout Medical City (SSMC), Abu Dhabi
5	Dr. Ahmed Abdulkareem Al Hammadi	Tawam Hospital, Al Ain
6	Dr. Alaa MM Enshasy	Dubai Health Authority, Dubai
7	Dr. Amal Mubarak Madhi	Abu Dhabi Public Health Center, Abu Dhabi
8	Dr. Anju Nabi	Dubai Academic Health Corporation (DAHC), Dubai
9	Dr. Anup Shashikant Poddar	Al Sharq Hospital, Fujairah
10	Dr. Arun Kumar Jha	Danat Al Emarat Hospital, Abu Dhabi
11	Dr. Ayesha Abdulla Al Marzooqi	Abu Dhabi Public Health Center, Abu Dhabi
12	Dr. Bashir Aden	Khalifa University, Abu Dhabi
13	Dr. Deeba Jafri	Purelab, Sheikh Khalifa Medical City, Ajman
14	Dr. Duckjin Hong	Sheikh Khalifa Specialty Hospital (SKSH) RAK
15	Dr. Farah Ibrahim Al-Marzooq	United Arab Emirates University, Al Ain
16	Dr. Fatima Al Dhaheri	United Arab Emirates University, Al Ain
17	Dr. Ghada Abdel Wahab	Abu Dhabi Agriculture and Food Safety Authority, Abu Dhabi
18	Dr. Ghalia Abdul Khader Khoder	University of Sharjah, Sharjah
19	Dr. Gitanjali Avishkar Patil	NMC Specialty Hospital, Abu Dhabi
20	Dr. Hafiz Ahmad	RAK Hospital, Ras Al Khaimah
21	Dr. Hazim Khalifa	Department of Veterinary Medicine, UAE University, Al Ain
22	Dr. Husein Alzabi	Sheikh Khalifa General Hospital, Um al Quwain
23	Dr. Ibrahim Alsayed Mustafa Alhashami	Purelab, Al Qassimi Hospital, Sharjah
24	Dr. Irfaan Akthar	Mediclinic City Hospital, Dubai
25	Dr. Jens Thomsen	Abu Dhabi Public Health Center, Abu Dhabi
26	Dr. John Stelling	WHONET, Boston, USA
27	Dr. Kavita Diddi	Prime Hospital, Dubai
28	Dr. Krishnaprasad Ramabhadran	Burjeel Hospital, Abu Dhabi
29	Dr. Laila Al Dabal	Dubai Academic Health Corporation (DAHC, Dubai)
30	Dr. Madikay Senghore	Khalifa University, Abu Dhabi
31	Dr. Manal Abdel Fattah Ahmed	PureLab, Ras Al Khaimah
32	Dr. Maya Habous	Rashid Hospital, Dubai Academic Health Corporation, Dubai
33	Dr. Moeena Zain	American Hospital Dubai
34	Dr. Monika Maheshwari	Al Zahra Hospital, Dubai
35	Dr. Monika Maheshwari	Medeor 24x7 Hospital, Dubai
36	Dr. Mubarak Saif Alfaresi	Zayed Military Hospital, Abu Dhabi
37	Dr. Mushtaq Khan	United Arab Emirates University, Al Ain
38	Dr. Najiba Abdulrazzaq	Al Kuwait Hospital, Emirates Health Services Establishment, Dubai
39	Dr. Nehad Nabeel Al Shirawi	Al Fujairah Hospital
40	Dr. Nesrin Helmy	Mediclinic Al Noor Hospital - Khalifa Street, Abu Dhabi
41	Dr. Prashant Nasa	NMC Specialty Hospital Al Nahda, Dubai
42	Dr. Rajeshwari T. A. Patil	Burjeel Medical City, Abu Dhabi
43	Dr. Ratna A. Kurahatti	NMC Royal Hospital Khalifa City A, Abu Dhabi
44	Dr. Riyaz Amirali Husain	Dubai Hospital, Dubai Academic Health Corporation, Dubai
45	Dr. Robert Lodu Serafino Wani Swaka	Sheikh Shakhbout Medical City, Abu Dhabi
46	Dr. Savitha Mudalagiriyappa	University Hospital Sharjah, Sharjah
47	Dr. Seema Oommen	Burjeel Medical City, Abu Dhabi
48	Dr. Shaikha Ghannam Alkaabi	Abu Dhabi Public Health Center, Abu Dhabi
49	Dr. Simantini Jog	Fakeeh University Hospital, Dubai
50	Dr. Simantini Jog	King's College Hospital London Dubai Hills, Dubai
51	Dr. Siobhan O‘Sullivan	Khalifa University, Abu Dhabi
52	Dr. Somansu Basu	NMC Specialty Hospital, Al Ain
53	Dr. Yassir Mohammed Eltahir Ali	Animal Wealth Sector, Abu Dhabi Agriculture and Food Safety Authority, Abu Dhabi
54	Dr. Yousuf Mustafa Naqvi	Department of Health Abu Dhabi (DoH), Abu Dhabi
55	Dr. Zulfa Omar Al Deesi	Latifa Maternity & Pediatric Hospital, Dubai
56	Emmanuel Fru Nsutebu	Sheikh Shakhbout Medical City, Abu Dhabi
57	Fouzia Jabeen	Purelab, Sheikh Khalifa Hospital, Abu Dhabi
58	Francis Amirtharaj Selvaraj	Sheikh Khalifa Medical City (SKMC), Abu Dhabi
59	Hadayatullah Ghulam Muhammad	Emirates International Hospital, Al Ain
60	Imene Lazreg	University of Sharjah, Sharjah
61	Kaltham Ali Kayaf	Ministry of Climate Change & Environment (MOCCAE), Dubai
62	Laura Thomsen	University of Freiburg, Germany
63	Leili Chamani-Tabriz	Clemenceau Medical Center, Dubai
64	Pamela Fares Mrad	Abu Dhabi Public Health Center (ADPHC), Abu Dhabi
65	Pascal Frey	Berne University Hospital, Berne, Switzerland
66	Prof. Abiola Senok	College of Medicine, Mohammed Bin Rashid University of Medicine and Health Sciences, Dubai
67	Prof. Agnes-Sonnevend-Pal	University of Pécs, Pécs, Hungary
68	Prof. Andreas Podbielski	University Hospital Rostock, Rostock, Germany
69	Prof. Carole Ayoub Moubareck	College of Natural and Health Sciences, Zayed University, Dubai
70	Prof. Dean Everett	Department of Pathology and Infectious Diseases, College of Medicine, Khalifa University, Abu Dhabi
71	Prof. Godfred A. Menezes	Department of Medical Microbiology and Immunology, RAK Medical and Health Sciences University, Ras Al Khaimah
72	Prof. Hala Ahmed Fouad Ismail	PureLab, Al Qassimi Hospital, Sharjah
73	Prof. Mohamud M. Sheek-Hussein	United Arab Emirates University, Al Ain
74	Prof. Peter Nyasulu	Department of Global Health, Faculty of Medicine and Health Sciences, Stellenbosch University, South Africa
75	Prof. Sameh Soliman	University of Sharjah, Sharjah
76	Prof. Tibor Pal	University of Pécs, Pécs, Hungary
77	Rania El Lababidi	Dept. of Pharmacy Services, Cleveland Clinic Abu Dhabi
78	Saeed Hussein	Erada Center for Treatment and Rehabilitation, Dubai
79	Stefan Weber	Purelab, Abu Dhabi
80	Sura Khamees Majeed	Al Gharbia Hospitals - Madinat Zayed Hospital
81	Syed Irfan Hussein Rizvi	Mediclinic City Hospital, Dubai
82	Timothy Anthony Collyns	Tawam Hospital, Al Ain
83	Zahir Osman Babiker	Sheikh Shakhbout Medical City (SSMC), Abu Dhabi

## Footnotes

^1^ https://whonet.org/

## Supplementary material

Supplementary Figure 1 Number of MRSA per annum and ratio per site (2010–2021).

Supplementary Table 1A Number and percentage of *Staphylococcus aureus* and methicillin resistant *S. aureus* (MRSA) isolates per Emirate.

Supplementary Table 1B Number of MRSA per year according to hospital unit.
