# Supplementary figures and images for: Methicillin resistant Staphylococcus aureus in the United Arab Emirates: a 12-year retrospective analysis of evolving trends
Source: Front Public Health. 2023 Dec 7;11:1244351. doi: 10.3389/fpubh.2023.1244351 (PMC10748512; doi:10.3389/fpubh.2023.1244351)

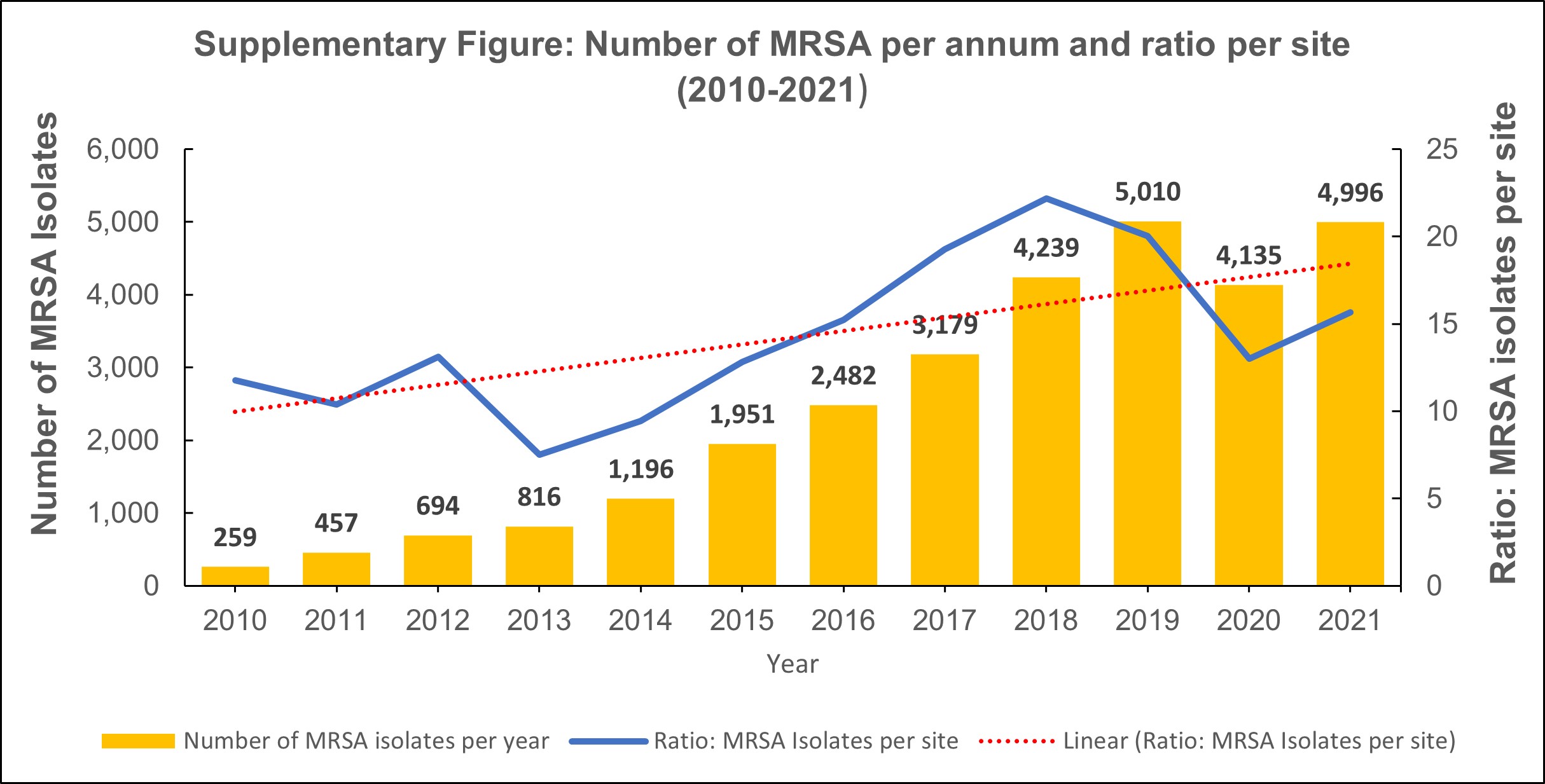

Supplement: Supplementary Figure 1 — Number of MRSA per annum and ratio per site (2010–2021). [file Image_1.jpg]
