# Supplementary material for: Methicillin resistant Staphylococcus aureus in the United Arab Emirates: a 12-year retrospective analysis of evolving trends
Source: Front Public Health. 2023 Dec 7;11:1244351. doi: 10.3389/fpubh.2023.1244351 (PMC10748512; doi:10.3389/fpubh.2023.1244351)
Supplement: Supplementary Table 1A — Number and percentage of Staphylococcus aureus and methicillin resistant S. aureus (MRSA) isolates per Emirate. [file Table_1.pdf]

Supplementary Table 1A: Number and percentage of *Staphylococcus aureus* and methicillin resistant *S. aureus* (MRSA) isolates per Emirate

	Total # of isolates (2010-2021)								
		Abu Dhabi	Dubai	Sharjah	Ras Al Khaimah	Ajman	Fujairah	Umm Al Quwain	Missing
All Staphylococcus aureus	111623	68845	27244	5788	4074	2415	1468	1742	47
MRSA	29414	18,036	6,483	1,916	1,221	771	516	471	
**Percentage MRSA**	**26.4**	**26.2**	**23.8**	**33.1**	**30.0**	**31.9**	**35.1**	**27.0**	**0.0**

Supplementary Table 1B: Number of MRSA per year according to hospital unit

	Total number of MRSA 2010-2021 (N=29,414)		
**Year**	**Inpatient**	**Outpatient**	**Unknown**
2010	134	123	2
2011	235	212	10
2012	334	301	59
2013	344	309	163
2014	424	358	414
2015	506	439	1006
2016	527	723	1232
2017	720	872	1587
2018	1116	1517	1606
2019	1222	1992	1796
2020	1129	1951	1055
2021	1562	2450	984
**Total**	**8253**	**11247**	**9914**
